# Acne tarda: Recommendations for classification, treatment and care as a result of an expert discussion

**DOI:** 10.1111/ddg.15913

**Published:** 2025-12-05

**Authors:** Matthias Augustin, Thomas Dirschka, Peter Arne Gerber, Martina Kerscher, Natalia Kirsten, Falk Ochsendorf

**Affiliations:** ^1^ Institute for Health Services Research in Dermatology and Nursing (IVDP) University Medical Center Hamburg‐Eppendorf (UKE) Hamburg Germany; ^2^ CentroDerm Clinic Faculty of Health University of Witten‐Herdecke Witten Germany; ^3^ Dermatologie am Luegplatz Düsseldorf Germany Department of Dermatology Medical Faculty Heinrich Heine University Düsseldorf Düsseldorf Germany; ^4^ Division of Cosmetic Sciences Faculty of Chemistry University of Hamburg Hamburg Germany; ^5^ Department of Dermatology Venereology and Allergology University Hospital Frankfurt Frankfurt Germany

**Keywords:** Acne, acne tarda, acne vulgaris, adult acne, consensus, late onset acne, recommendations, retinoids, treatment

## Abstract

Acne tarda is defined in the literature as adult acne, which according to most authors occurs in women aged 25 and older. However, the definitions and age groups vary depending on the study. Current guidelines rarely address adult acne. In this review, current studies and the literature on acne tarda are analyzed and evaluated by German experts. Recommendations regarding classification, clinical features, differentiation, and treatment of acne tarda were summarized in a consensus based on the discussion. The recommendations also include the treatment of post‐inflammatory erythema/hyperpigmentation and acne scars, as well as the accompanying skin care. The goal is to improve the treatment of patients with acne tarda.

## INTRODUCTION

Acne vulgaris, an inflammatory disease of the sebaceous gland follicle, is one of the most common skin diseases worldwide, with an increasing incidence in some countries over the last 20 years.^1^ Although the prevalence in Germany has remained stable in recent years, the age‐standardized prevalence rate was 5% in 2019, ranking the country among the top 20 worldwide.^1^ Acne is most common in adolescents, with a prevalence of up to 85% between the ages of 15 and 18.^2^ Acne places a high psychological and social burden on many affected individuals, leading to a severe loss of quality of life and potentially resulting in depression and an increased risk of suicide.^3^, ^4^, ^5^

Acne vulgaris includes acne comedonica (comedones predominate), mild to moderate acne papulopustulosa (inflammatory papules and pustules predominate), severe acne papulopustulosa/moderate acne nodularis (nodules and cysts present) as well as severe nodular acne and acne conglobata (abscessing nodules).^2^, ^5^ Acne tarda is predominantly defined in the literature as acne that persists beyond the age of 25 (persistent acne) or appears for the first time or again after this age.^2^, ^6^, ^7^

To date, no fundamental differences in the causes and course of acne tarda compared to adolescent acne vulgaris have been identified.^8^, ^9^ Although a strong influence of genetic factors was assumed in a twin study with adult female twins, there are still many unanswered questions about the pathogenesis of acne tarda.^10^

In addition to the variable definition of acne tarda and the insufficiently investigated pathogenesis, its specific treatment is also not subject to any consensual therapy recommendations. The latest national and European guidelines for the treatment of acne vulgaris were published 7–13 years ago, but their treatment recommendations referred to acne in general.^5^, ^11^, ^12^ With regard to acne tarda, the German guideline only recommends hormone tests.^11^ Further points, such as treatment of comorbidities, psychosocial diagnostics, and special features of topical basic therapy, are rarely addressed.

In this context, dermatology experts from Germany reviewed, discussed, and evaluated the current literature on acne tarda during a virtual working meeting. Based on an initial written summary, ambiguities were discussed in a further virtual conference and summarized in a consensus or formulated as open questions. The wording for the strength of recommendation (strong recommendation: “should [not]”, recommendation: “ought [not] to”, open recommendation: “can be considered/waived”) was confirmed by all experts. Finally, existing treatment recommendations for acne vulgaris were evaluated in the second meeting and supplemented with expert experiences for acne tarda. These recommendations are intended to provide those affected by acne tarda with faster access to appropriate therapies and accompanying skin care.

## DEFINITION OF ACNE TARDA

Acne tarda, also known as late onset acne, adult acne or post‐adolescent acne, is predominantly defined in the literature as acne that occurs beyond the age of 25.^2^, ^6^, ^7^ However, the definition of acne tarda by age is variable, as some textbooks, observational and clinical studies also define acne tarda from an earlier age of 18 to 20 years (online supplementary Table S1). In review articles, the definition “age of ≥ 25 years” is usually used, or acne tarda is generally referred to as a subtype of adult acne in women, which occurs for the first time at the beginning to middle of the third decade of life.^8^ For clinical studies, the definition of adult in the legal sense (≥ 18 years) is often used and the terms “adult acne” and “adult acne vulgaris” are mainly used, whereby the age of the patients included varied most frequently. In addition, acne tarda is often treated in the literature exclusively as a disease of women (online supplementary Table S1). This may be due to the frequently reported higher prevalence of adult acne in women, which, however, is not clearly proven according to a systematic analysis of the literature.^13^

Some studies differentiate acne tarda into two subtypes, but with different criteria and different nomenclature: most frequently, persistent or recurrent acne tarda is differentiated from late‐onset acne tarda.^14^, ^15^, ^16^, ^17^ In their observational study of 226 female patients with postadolescent acne, Capitanio et al. differentiated between the clinical characteristics of “comedonal postadolescent acne” (85% of those affected) and “papulopustular postadolescent acne” based on their clinical appearance.^18^ The more common comedonal form was associated with a late first appearance of acne.^18^ A similar distinction between “non‐inflammatory” and “inflammatory” forms is also used by Jansen and colleagues in a review article.^6^ To summarize, the definitions of acne tarda, the associated age and sex of those affected differ significantly in the literature.

### Expert discussion on “Definition of acne tarda”

The experts agreed on the following findings from the literature and their own experience:

- The term “acne tarda” should be used as a synonym for adult acne/late acne/post‐adolescent acne.

According to textbook definitions, experts agree that acne tarda is not limited to a first manifestation of acne in adulthood but also includes persistent or recurrent acne beyond the age of 25. To ensure that the condition does not merely represent a self‐limited case persisting from puberty, acne tarda should continue to be defined as post‐adolescent acne occurring at ≥ 25 years of age.

- By definition, acne tarda should include both sexes.

According to experts, acne tarda can affect both men and women. According to an Indian study, there are no clinical differences in acne tarda characteristics between men and women.^19^ The authors tended to observe nodules and cysts more frequently in women, while acne tarda in men more frequently manifested itself similarly to classic acne vulgaris. In women, the hormonal influence should also be taken into account, specific questions should be asked about the use or discontinuation of oral contraceptives and, if necessary, a hormone analysis should be considered in order to rule out endocrinopathies such as polycystic ovary syndrome (PCOS) or adrenogenital syndrome (AGS) (see “Concomitant/associated diseases and differential diagnoses”).

- In addition to age, acne tarda can be defined by its clinical appearance.

There are some fundamental clinical differences between adolescent acne vulgaris and acne tarda, which can also guide the diagnosis of acne tarda (see “Clinical relevance and characteristics of acne tarda**”**).

## PREVALENCE OF ACNE TARDA

**FIGURE 1:**
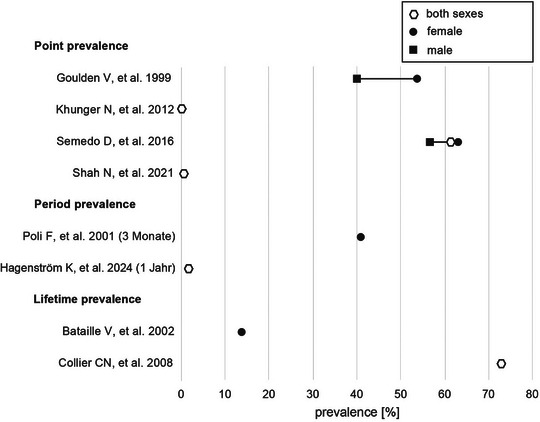
Prevalences of the studies listed in Table 1 with a focus on acne tarda. Plotted according to prevalence type and gender, if indicated. Case numbers were not taken into account.

With prevalence rates varying between 0.4 and 73.3% depending on the study, acne tarda is much more common on average than many dermatologists assume (Table 1, Figure 1). The wide variation is mainly due to different data sources and data collection methods, non‐standardized study designs, diagnostic criteria, and observation periods as well as center effects.

**TABLE 1:** Prevalence of acne tarda. Literature review of observational studies and self‐assessment focusing on adult acne.

Publication	Country	Study type	Age [years]	Study population (n)	Prevalence	Remark
***Point prevalence***						
Cunliffe WJ, Gould DJ. 1979^20^	Great Britain	Survey, physical examination	18–70	2133; 1,162 ≥ 25	Approximately 30–40% physiological acne in women aged 24	Higher prevalence in women aged 24 and over; prevalence decreases with increasing age
Stern RS. 1992^21^	USA	Re‐evaluation of dermatological examination (NHANES study)	15–44	20,749	27% (women), 34% (men)	Higher prevalence in men; adolescent acne also recorded
Goulden V, et al. 1999^22^	Great Britain	Random selection and examination at the hospital and surrounding area	≥ 25	749	48%	Higher prevalence in women (54% vs. 40% in men); 80% persistent acne
Schäfer T, et al. 2001^23^	Germany	Standardized interview and dermatological examination	1–87	896 (20–87 years: 761)	26.8% (20–87 years: 26.7%)	Higher prevalence in men (29.9% vs. 23.7% in women); correlation with smoking; also includes adolescent acne
Augustin M, et al. 2011^24^	Germany	Full‐body dermatological examination at the workplace	16–70	90,880	3,9%	Proportion of men/women balanced; also includes adolescent acne
Khunger N, et al. 2012^19^	India	Proportion of acne patients in dermatological clinics	≥ 25	72,710	0,38%	Prevalence calculated in relation to patients with other skin diseases; higher prevalence in women; 73.2% persistent acne
Semedo D, et al. 2016^25^	Portugal	Questionnaire and physical examination	20–60	1,055	61,5%	Mild acne only detected by clinical examination; no difference in prevalence between men and women
Shah N, et al. 2021^26^	India	Dermatological examination	≥ 25	24,056	0,74%	Unclear study population; higher prevalence in women
Kirsten N, et al. 2021^27^	Germany	Whole‐body dermatological examination in 500 German companies	≥ 16	161,269	3,3%	Highest prevalence 16–29 years (9.9%); Decreasing prevalence with increasing age; also includes adolescent acne
***Period prevalence***						
Poli F, et al. 2001^28^	France	Questionnaire to 4000 adult women; period prevalence (3 months)	25–40	3305	41%	Self‐assessment, only women included
Thyssen JP, et al. 2022^29^	Denmark	Use of nationwide administrative data; period prevalence (1 year)	12–≥ 40	66,000	3,7%	Control cohort; proportion of women with acne slightly increased; also includes adolescent acne
Hagenström K, et al. 2024^30^	Germany	Performance data analysis (DAK‐Gesundheit); period prevalence (1 year, determined for the years 2016–2020)	≥ 25 years and ≥ 30 years	≥ 25: depending on the year 1,880,000–2,040,000 ≥ 30: depending on the year 1,770,000–1,900,000	≥ 25: 1,5–1,7% ≥ 30: 1,3–1,4% (Rates standardized by age and gender)	Proportion of women increased (2020: 73.8% vs. 26.2%)
***Lifetime prevalence***						
Bataille V, et al. 2002^10^	Great Britain	Female twin study, questionnaire	18–79	3,114	14%	Retrospective self‐assessment; women only, genetic link established
Collier CN, et al. 2008^31^	USA	Questionnaire to university and hospital	≥ 20	1,013	73,3%	Self‐assessment of ever having had acne (even as a teenager); higher prevalence ≥ 20 years observed in women

*Abbr*.: NHANES, National Health and Nutrition Examination Survey; n/a, not specified

The literature also reports that a higher proportion of male adolescents are affected by adolescent acne, while proportionally more women report acne tarda.^22^, ^26^, ^31^ This assumption was not confirmed in prevalence studies that analyzed acne tarda on a gender‐specific basis (Figure 1) and has also been discussed controversially in the literature.^13^, ^21^, ^23^

### Expert discussion on “Prevalence of acne tarda”

- To determine the exact prevalence of acne tarda and its subtypes, further studies ought to be carried out according to standardized inclusion and diagnostic criteria.

Previous studies to determine the prevalence of acne tarda are often not comparable as no standardized inclusion and diagnostic criteria were used. The experts would welcome a study in German‐speaking countries that uses standardized criteria. They recommend considering the following aspects:

- A representative, population‐based study cohort
- Inclusion of both women and men aged ≥ 25 years
- Standardized assessment of acne tarda subtypes, severity on the face and trunk, localization, and treatment response
- Evaluation of subjective stress using validated patient‐reported outcomes (PROs)
- Hormonal assessment for all participants
- Evaluation of treatment response over a period longer than the usual 12 weeks

Such a study would provide a more comprehensive overview of the situation in Germany.

## CLINICAL RELEVANCE AND CHARACTERISTICS OF ACNE TARDA

Acne tarda can have a greater impact on the quality of life of older patients than acne vulgaris in younger people.^8^, ^32^ Accordingly, a study based on *Skindex 29 questionnaires* showed that adult acne patients experienced similar emotional and functional impairments as psoriasis patients despite a significantly lower symptom burden.^32^ Central points of acne‐related emotional effects are reduced self‐esteem, poor self‐image, low self‐confidence and feelings of shame, which are often associated with a decrease in social interactions.^33^ The impairment in quality of life correlates with the severity of acne tarda, and the psychological effects are still often underestimated.^8^, ^15^, ^32^ This makes treatment with suitable, effective therapies all the more important, as this can improve the patient's quality of life as well as their appearance.^8^, ^34^, ^35^

According to the literature, two clinical types of acne tarda can be distinguished:

1. Non‐inflammatory acne tarda (acne comedonica tarda), characterized by large, closed, or cyst‐like comedones with a low proportion of inflammatory lesions (< 5%)
2. Inflammatory acne tarda (acne papulopustulosa tarda/acne papulopustulo‐nodosa tarda), manifesting as papules, pustules, and deep inflammatory nodules (proportion of inflammatory lesions >5%) that frequently lead to scarring.^6^, ^36^

**FIGURE 2:**
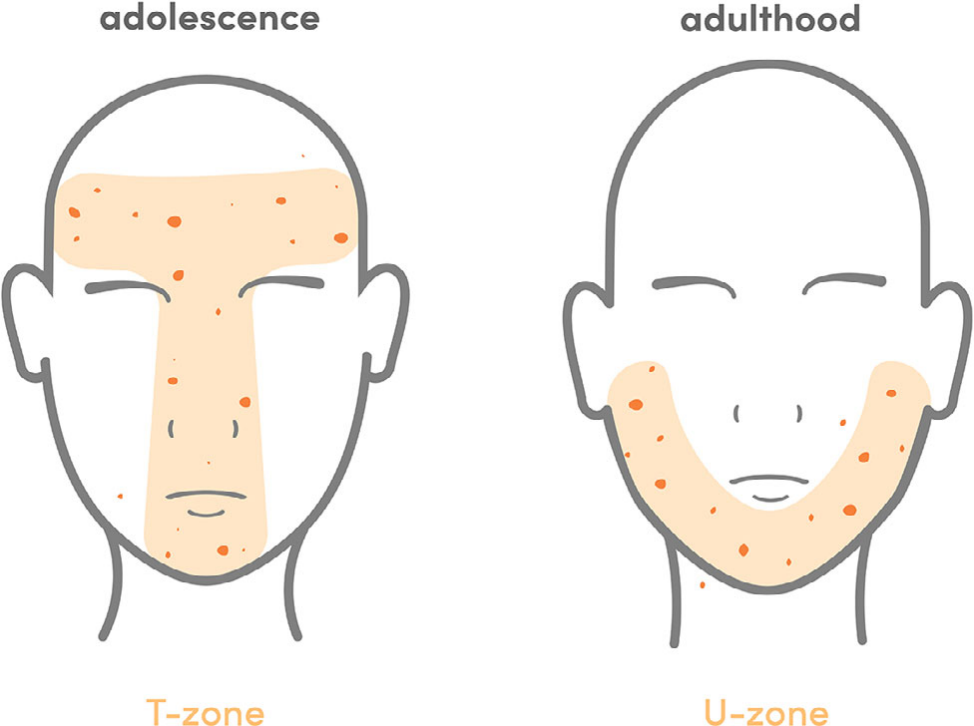
Differences in acne localization in adolescents (T‐zone: forehead, nose, upper chin area) and adults (U‐zone: cheeks, perioral, lower chin area) (Adapted from Dréno et al.^8^)

In fact, different localizations of acne efflorescence have been found depending on age. While papules, pustules and comedones occur more frequently in the T‐zone of the face (forehead, nose, upper chin area) and on the trunk in adolescent acne vulgaris, the U‐zone (cheeks, perioral, lower chin area) is more likely to be affected in adults (Figure 2).^8^, ^37^ These differences could also indicate a separate pathomechanism in each case.^8^ Furthermore, in contrast to acne vulgaris, acne tarda often requires long‐term therapy and may lead to increased scarring.^37^, ^38^, ^39^

To summarize, adolescent acne vulgaris and acne tarda differ in some clinical characteristics. However, due to the variable definition of acne tarda, clear classification is difficult.

### Expert discussion on “Clinical relevance and characteristics of acne tarda”

Based on the literature, the clinical features of acne vulgaris and acne tarda subtypes were summarized in Table 2 and supplemented by the experts' own experiences.

- A distinction should be made between post‐inflammatory erythema (PIE)/post‐inflammatory hyperpigmentation (PIH) and acne scars.

**TABLE 2:** Comparison of adolescent acne vulgaris and acne vulgaris tarda. In the case of acne tarda, characteristics of female acne tarda were also included.

Adolescent acne vulgaris	Acne tarda (post‐adolescent acne)	
	*Non‐inflammatory acne tarda/acne comedonica tarda*	*Inflammatory acne tarda/acne papulopustulosa tarda/acne papulo‐pustulo‐nodosa tarda*
Most common during puberty (between 15–18 years)	≥ 25 years	
Comedones, papules, pustules^37^	Comedones, inflammatory lesions less than 5%^38^	No comedones, inflammatory lesions > 5%, papules, pustules, and nodules^6^, ^36^, ^38^
	Rather persistent/recurrent from adolescence (expert judgement) The more common form of acne tarda, accounting for approx. 60‐80% of cases^15^, ^18^, ^22^, ^28^	Also, first manifestation in adulthood (expert judgement)
Rapid onset in severe form^37^	Gradual onset with mainly mild/moderate manifestation^6^, ^14^, ^37^	
Increased in the T‐zone of the face^8^, ^14^, ^37^	Distributed, preferably cheeks, forehead^6^	Increased in the U‐zone of the face (cheeks, perioral, lower chin area)^6^, ^37^
Less scarring in comparison, depending on the degree of severity^39^	Scarring observed only with persistent form^18^	Increased scarring, boxcar scars or “rolling scars” (deeply indented scars)^18^
Good response to therapy^38^	Better response to therapy than acne papulopustulosa tarda/acne papulo‐pustulo‐nodosa tarda (expert judgement)	Long‐term therapy necessary, frequent relapses^6^, ^9^, ^37^, ^38^, ^39^
Comparable sebum excretion rates and *Cutibacterium acnes* colonisation^14^		

It is generally reported that acne tarda can lead to increased scarring (Table 2). It is assumed that this is directly related to the increased proportion of inflammatory lesions and persistent acne.^39^ The most common acne scars are atrophic scars.^40^ In these lesions, inflammation induces increased expression of collagen‐ and elastin‐degrading enzymes such as matrix metalloproteinases (MMPs) and elastase, which leads to dermal tissue breakdown. An imbalance between MMPs and their regulators, the tissue inhibitors of metalloproteinases (TIMPs), results in a net loss of tissue.^41^

In addition, post‐inflammatory erythema (PIE) or post‐inflammatory hyperpigmentation (PIH) can occur, also as a result of self‐damage to the skin (dermatitis factitia).^42^ PIE/PIH should be differentiated from acne scars: PIE is caused by vasodilation of the superficial skin vessels, while PIH is due to melanogenesis stimulated by inflammation, in which the melanin released is deposited in the form of melanosomes either directly in the keratinocytes of the epidermis or via uptake by macrophages in the dermis.^43^ If left untreated, PIE can last for several months, while PIH – depending on the cause, severity of the inflammation and skin type – can persist for months to years or even decades.^44^ In contrast to acne scars, PIE/PIH are not tissue defects, which is why different therapeutic approaches are required for successful treatment (see “Therapy standards in practice”).

## AETIOPATHOGENESIS

No clear differences in the pathogenesis of acne tarda compared with adolescent acne vulgaris have been identified.^8^, ^9^ However, the significance of the individual pathogenetic factors in acne tarda could be assessed differently.^6^ Basically, the development of acne is based on a combination of four pathogenetic factors: *(1)* increased sebaceous gland activity with increased sebum production (seborrhea); *(2)* abnormal follicular differentiation with increased keratinization (keratinization disorder); *(3)* microbial overgrowth with *Cutibacterium acnes* in the pilosebaceous unit; *(4)* intra‐ and perifollicular inflammation.^17^, ^45^ Inflammation is a central pathogenetic factor, as acne lesions can also develop without clinically visible comedones and inflammation can be detected histologically even before the visible development of an acne lesion.^46^, ^47^ In patients with acne tarda, seborrhea and increased androgen secretion were found to be comparable to acne vulgaris. Other inflammation‐related signaling pathways, genetic predisposition, and external triggers (e.g., diet, stress, medication, smoking) are also being discussed as underlying factors.^6^, ^8^, ^9^, ^45^ For example, 37% of patients with acne tarda (defined as ≥ 25 years) were found to have symptoms of hyperandrogenemia, while in another study smoking was particularly associated with the comedonal acne tarda type.^6^, ^18^

Sebum production has been shown to be stimulated by the release of IGF‐1 (insulin‐like growth factor 1) and androgens (testosterone, dihydroepiandrosterone sulphate [DHEA‐S]).^17^ For example, a positive correlation of increased IGF‐1 and DHEA‐S levels was found in adult women with acne tarda, but not in adult men.^48^ However, no correlation between androgenic hormone serum concentrations and acne was found in other studies.^37^, ^49^ As patients with acne often report the appearance or disappearance of acne lesions in connection with menstruation and pregnancy, there is a consensus that there is a hormonal influence on acne.^6^, ^8^, ^37^ However, the extent to which this is relevant in individual cases remains unclear.

A genetic predisposition to acne tarda is likely, as more than half of affected individuals report first‐degree relatives with the condition, and the risk is increased in the presence of a positive family history.^50^ In a twin study of more than 1,500 adult female twins (458 monozygotic, 1,099 dizygotic), those affected by acne reported a positive first‐degree family history significantly more often.^10^ In addition, genetic modelling attributed 81% of the acne‐specific population variance to genetic effects. The remaining 19% was attributed to individual environmental factors.^10^

While no genetic studies specific to acne tarda have been published, genome‐wide sequencing techniques (next generation sequencing, NGS) have identified acne vulgaris‐associated susceptibility loci in genes of the TGF‐β (transforming growth factor‐β) signaling pathway (*OVOL1*, *FST*, *TGFB2*).^51^ A decrease in *OVOL1* and *TGFB2* expression in inflammatory acne papules compared to healthy skin of the same patients was also confirmed.^51^ In a second and third follow‐up study, a total of 43 loci were identified that are associated with an increased susceptibility to acne with disorders in the development, morphology and activity of the sebaceous follicle (including *WNT10A*, *EDAR*, *LGR6*, *TP63*, *MANC2*).^52^, ^53^ Only a missense mutation of *WNT10A* showed a higher effect size in men than in women; all other genes identified showed no gender‐specific differences.^52^ Interestingly, the associated genes are partly linked to ectodermal dysplasias (*WNT10A*, *TP65*, *EDAR*) and neutrophilic autoinflammatory dermatoses (Generalized Pustular Psoriasis, GPP: *IL36RN*), highlighting the importance of hair follicle structure and morphology in dermatological diseases.^52^, ^53^ These studies were conducted in cohorts of European ancestry. However, gene associations identified in Chinese studies could not be reproduced, suggesting ethnic differences in the genetic factors contributing to acne.^52^, ^53^ Overall, processes that contribute to the development and maintenance of the hair follicle are thus potential new therapeutic targets in addition to the current therapeutic regimen, which aims to regulate keratinization disorder, inflammation, and bacterial colonization.

### Expert discussion on “Aetiopathogenesis”

Many basic concepts of acne etiopathogenesis have been known for years. However, given the high prevalence of acne and its considerable psychosocial impact, surprisingly little fundamental research has addressed the exact mechanisms underlying its development. The experts therefore formulated the following open questions on the causes and course of acne tarda:

- It is assumed that there are around 400–900 sebaceous gland follicles per cm^2^ on the face. Even with moderate to severe acne, only a small percentage (0.25%) are involved in visible lesions.^54^ Why?
- The gene association studies carried out to date explain around 6% of the variance in acne risk.^53^ This means that the familial correlations of acne remain largely unexplained and further studies are needed in this regard.
- What pathomechanistic differences underlie the deviating acne localization in adolescents (T‐zone) vs. adults (U‐zone)?
- What role does the microbiome play in the development/maintenance of the inflammatory reaction?
- Can the analysis of biomarkers enable personalized treatment of acne tarda in the future?
- How great is the influence of lifestyle factors and how reliable are patient consultations in this regard?

## CONCOMITANT/ASSOCIATED DISEASES AND DIFFERENTIAL DIAGNOSES

One of the most common comorbidities associated with acne tarda is polycystic ovary syndrome (PCOS), which is associated with acne in around 30% of the cases.^2^ Conversely, it has been shown that around 20% of adult women with acne tarda had PCOS.^49^ It is characterized by hyperandrogenemia, insulin resistance, oligo‐ and anovulation and polycystic ovaries. Acne, hirsutism, and androgenetic alopecia are typical concomitant symptoms of PCOS and should always be clarified by differential diagnosis in patients with acne tarda.^2^ Other syndromes that are frequently associated with acne and are accompanied by hyperandrogenemia, insulin resistance or autoinflammation are Hyperandrogenism, insulin resistance and acanthosis nigricans (HAIR‐AN) syndrome, adrenogenital syndrome (AGS or congenital adrenal hyperplasia [CAH]).^2^

Psychosomatic and psychiatric comorbidities (depression, social anxiety, body dysmorphic disorder, obsessive‐compulsive disorder) are also frequently associated with acne vulgaris.^55^ These affect both adolescent and adult patients with acne, who often withdraw from social activities such as going out, sports and restaurants visits.^56^ The psychological burden of acne disease should therefore not be neglected and should be considered in patient care.^8^

Overall, concomitant diseases should be recognized and, if necessary, considered in a holistic treatment regimen. It is also necessary to differentiate acne tarda from dermatoses with a similar clinical appearance. Table 3 lists the differential diagnoses and their distinguishing features.

**TABLE 3:** Differential diagnoses of acne tarda and their distinguishing features.

Differential diagnosis	Clinical differentiation features
Acne excoriée (des jeunes filles)	Polymorphic lesions due to constant manipulation, often hyperpigmented, hemorrhagic crusted excoriations, shallow ulcerations that heal slowly^2^
Acne cosmetica (cosmetic acne)	Dense, predominantly closed comedones and isolated papulopustules on forehead, cheeks, perioral region; triggered by cosmetics^2^
Mechanical acne, tar acne, chloracne	Triggered by pressure points/rubbing, contact with tar, poisoning by hydrocarbon compounds
Perioral and periorbital dermatitis	Mainly localized around the mouth or eye area^8^
Gram‐negative folliculitis	Predominantly centrofacial, follicular pustules on reddened skin, severe seborrhoea^8^, ^17^
Pityrosporum folliculitis	Hair follicle inflammation with *Pityrosporum ovale* (zoophilic, lipophilic yeast fungus)^8^, ^17^
Milia	Small keratin‐containing cysts; pearly white bumps directly under the skin surface; usually occur in multiples^8^
Rosacea (Pyoderma faciale)	Absence of comedones, usually no seborrhea, onset in the central facial area, eye involvement, sebaceous gland hyperplasia, often associated with telangiectasia

### Expert discussion on “Concomitant/associated diseases and differential diagnoses”

In addition to differentiating between the differential diagnoses, the experts recommend the following examinations when a patient presents with acne tarda:

- Women with acne tarda ought to be examined for hormonal disorders (PCOS, hyperandrogenemia). Dermatologists can carry out the initial diagnosis including the relevant laboratory tests. In the event of a positive finding for PCOS or if other endocrinological disorders are suspected, a referral should be made to an endocrinologist/gynecologist.
- A careful medical history and physical examination that goes beyond the detection of acne ought to be considered to identify clinical signs of hyperandrogenemia (such as menstrual irregularities, hair pattern).

## THERAPY STANDARDS IN PRACTICE

In general, therapy standards and guidelines that have been developed for acne therapy in adolescence also remain valid for acne tarda.^5^, ^6^, ^12^, ^57^ In addition, treatment adherence is essential for successful therapy.^8^ However, the current German S2k guideline “Therapy of acne” was developed over 10 years ago, and the latest update of the European S3 guideline was 7 years ago, which is why current therapy options for acne could not be taken into account in the therapy regimen.^5^, ^12^, ^55^ The guidelines of the *National Institute for Health and Care Excellence* (NICE) or the American guidelines for the treatment of acne vulgaris do not explicitly mention acne tarda either.^57^, ^58^ Although clinical studies specifically on acne tarda exist, they usually include only small study groups or represent post hoc analyses of acne vulgaris trials (supplementary Table S2). Severe acne is rarely considered here and acne papulo‐pustulosa‐nodosa tarda is generally excluded from the studies (supplementary Table S2).

The aim of acne therapy is to reduce inflammation quickly and sustainably, eliminate comedones or prevent new ones from forming, avoid scarring and improve the quality of life of those affected by selecting a therapy appropriate to the stage of the disease. Since as many pathogenetic factors as possible should be influenced simultaneously, a combination of several substances with additive or synergistic effects, preferably fixed combinations (adapalene/benzoyl peroxide [BPO], tretinoin/clindamycin, clindamycin/BPO) is recommended.^2^, ^57^, ^58^

### Expert discussion on “Therapy standards in practice”

Based on the recently updated American guidelines, the experts recommend a holistic therapy concept, which has been supplemented by their own experience and also takes skin care into account (Tables 4, 5).^57^

**TABLE 4:** Treatment recommendations for acne tarda. The recommendations are based on the American guideline for the treatment of acne vulgaris in adults, adolescents and prepubescents (≥ 9 years)^57^ and have been supplemented by expert experience.

	Guideline recommendation	Special features of acne tarda (expert experience)
First choice (clear recommendation of the US guideline)	**Mild**	
	Topical treatment (multimodal combination recommended): ‐ Topical retinoids (trifarotene, adapalene, tazarotene, tretinoin, isotretinoin) ‐ BPO ‐ Topical antibiotics (no monotherapy, only recommended in combination)	Topical retinoids, azelaic acid and BPO can be used permanently (maintenance therapy)
	Topical fixed combinations: ‐ Topical antibiotics + BPO (e.g., clindamycin 1% / BPO 5%) ‐ Topical retinoids + BPO (e.g., adapalene 0.1% / BPO 2.5%; adapalene 0.3% / BPO 2.5%) ‐ Topical retinoids + antibiotics (e.g., tretinoin 0.025% / clindamycin 10%) (simultaneous use of BPO can prevent the development of antibiotic resistance)	
	**Moderate/Severe** (Limitation of antibiotic therapy to avoid the development of resistance; combination with topical therapy (except for a combination with topical antibiotics)	
	Systemic antibiotics^*^: ‐ Doxycycline (preferred over azithromycin)	
	Hormonal treatment: ‐ Intralesional corticosteroids (adjuvant treatment for larger acne papules or nodules, if there is a risk of scarring, or for rapid improvement of inflammation and pain)	
	Isotretinoin^**^: Isotretinoin (preferably daily; lidose‐isotretinoin or standard isotretinoin	Set isotretinoin indication more generously to induce remission Use isotretinoin especially for nodules (to avoid scarring), if the effect on nodules is insufficient, intralesional corticosteroid treatment 1st attempt at withdrawal depending on clinical response (from about 6 months)
Second‐line therapy (recommendation of the US guideline with reservations)	**Mild**	
	Topical treatment: ‐ Clascoterone ‐ Salicylic acid ‐ Azelaic acid	
	**Moderate/Severe**	
	Systemic antibiotics^*^: ‐ Minocycline ‐ Sarecycline	
	Hormonal treatment: ‐ Combined oral contraceptives ‐ Spironolactone	Hormonal treatment for women only Spironolactone superior to doxycycline and well tolerated according to current study^59^ Desire to have children: azelaic acid, BPO, salicylic acid^60^

Abbr.: BPO, benzoyl peroxide.

* Limit use of antibiotics to reduce the risk of resistance development and other antibiotic‐associated complications; combination with BPO or other topical treatments recommended.

** Isotretinoin: patients with psychosocial distress or scarring should be considered as candidates for isotretinoin. Monitoring of liver function and lipids. Population‐based studies have not found an increased risk of neuropsychiatric disorders or inflammatory bowel disease with isotretinoin. Reliable, continuous contraception is mandatory for women of childbearing age.

**TABLE 5:** Further measures regarding dermocosmetics and the treatment of PIE/PIH/acne scars.

	**Further measures regarding acne tarda (expert experience)**
Dermocosmetics	Facial cleansing Medical cosmetics Superficial, chemical peelings Microdermabrasion *In general*: moisturizing care, UV protection
Treatment of PIE/ PIH/ acne scars	**PIE/ PIH**: Laser treatment not recommended **Acne scars**: Retinoids (trifarotene, adapalene, tretinoin) Chemical peelings Dermabrasion (Radio frequency) microneedling Laser (see S2k guideline “Laser therapy of the skin”)^61^

*Abbr*.: PIE, post‐inflammatory erythema; PIH, post‐inflammatory hyperpigmentation

Accordingly, topical retinoids are the treatment of choice for mild forms of acne tarda (especially acne comedonica tarda), both for the initiation of treatment and as maintenance therapy (Table 4). Multimodal combinations are recommended; whereby topical fixed combinations can also be used. For moderate to severe acne, systemic antibiotics of the tetracycline class (especially doxycycline) and intralesional corticosteroid injections (e.g., triamcinolone) are also recommended for the treatment of larger acne papules or nodules. Experts are also in favor of treatment with isotretinoin, particularly in the case of nodules or protracted acne tarda. Tetracycline antibiotics and isotretinoin are contraindicated in pregnancy, which is why the experts recommend macrolide antibiotics or topical BPO or azelaic acid if the patient wishes to have children (Table 4).

The American guideline did not contain any recommendations for light or laser therapies.^57^ 1,726 nm laser, whose wavelength specifically targets the sebaceous gland, is a potential future treatment option for moderate to severe acne.^62^, ^63^ However, due to the novelty of the method and the limited data available, neither the American guideline nor the expert panel make a recommendation in favor of this method.

Further comments and discussion by the experts on the therapy algorithm:

- Dermocosmetics can play an important role in the treatment of acne tarda.

Treatment with topical retinoids, BPO or their combination with other active ingredients or oral isotretinoin often leads to skin irritation, redness and tension.^64^ Dose adjustments can be made in an attempt to prevent these adverse events. Nevertheless, dermocosmetics/lipid‐replenishing skin care can also play an important role in reducing side effects and improving overall therapy adherence.^65^, ^66^ For example, the use of a non‐greasy, non‐comedogenic, fragrance‐free product showed a significant improvement in skin dryness, roughness, scaling and skin discomfort/tension after oral isotretinoin or topical tretinoin.^64^ Accordingly, in a cross‐sectional study, the use of accompanying dermocosmetics correlated with increased adherence to therapy.^67^ Other positive factors influencing adherence were acne severity, clinical improvement, patient satisfaction and patient education.^67^

Dermocosmetic routines should always be carried out in the morning and evening in the order of cleansing, moisturizing, and sun protection (only in the morning). In general, gentle cleansers that maintain the skin's pH (salicylic gels) and non‐comedogenic moisturizers that support the barrier repair process are recommended.^68^ As the integrity of the skin barrier is impaired in patients with acne tarda,^69^ which can be exacerbated by retinoid treatment, adequate UV protection (at least SPF 50) is also necessary.^65^ Non‐comedogenic products should also be used here. Medical cosmetics (specialized skin care products with active ingredients, as opposed to purely cosmetic products that primarily address surface appearance) may be considered. In the evening, facial cleansing should be repeated. Dermocosmetics should also be individually tailored to the patient's needs, like acne tarda therapy (Table 5).^65^

- A comprehensive therapy concept also includes the prevention and treatment of post‐inflammatory erythema (PIE), post‐inflammatory hyperpigmentation (PIH) and acne scars.

According to the experts, laser treatment should not be carried out for PIE. Post‐inflammatory hyperpigmentation does not respond to laser therapy and, if necessary, should be managed with pharmacological treatment. Acne therapies can have an effect on PIH or scars. Treatment with adapalene (0.3%) in combination with BPO (2.5%) led to a reduction in the formation of atrophic scars.^70^ In addition, reduced scarring was confirmed in a recent multicenter, vehicle‐controlled study on trifarotene.^71^ The results showed a significant continuous reduction of atrophic scars (2–4 mm) from an average of 11.4 scars by –5.9 scars on the trifarotene side vs. –2.7 on the vehicle side.^71^ Accordingly, retinoids can be recommended for the prevention and treatment of acne scars before resorting to chemical peels (e.g., trichloroacetic acid [TCA] peel), dermabrasion or laser. For atrophic (acne) scars, the S2k guideline “Laser therapy of the skin” recommends non‐ablative fractionated lasers such as the Er:Glass laser, dye laser (pulsed) or IPL (Intense pulsed light) for erythematous aspects of the scar, as well as ablative, fractionated CO_2_ or Er:YAG lasers (Table 5).^60^

- The success of the therapy can also be determined by a measurable improvement in the patient's quality of life.

According to experts, acne‐specific questionnaires on quality of life (e.g., Acne Disability Index (ADI), Dermatology Life Quality Index [DLQI]) can be helpful in individual cases to record quality of life (DLQI) or also the psychological impact of the disease (ADI) and thus justify certain treatment decisions.

## CONCLUSIONS

This review and consensus discussion aims to summarize current studies and literature on acne tarda and, together with the experts’ own expertise, to derive specific recommendations for its classification and treatment. The experts are in favor of the definition of acne tarda from the age of 25, which can affect both men and women. Clinically, a distinction was recommended between two subtypes, acne comedonica tarda and acne papulopustulosa tarda/acne papulo‐pustulo‐nodosa tarda. These differ mainly in the number of comedones and inflammatory lesions as well as in the expected response to therapy (Table 2). With regard to the pathogenesis of acne tarda, experts believe that further studies are needed to clarify the numerous unanswered questions. Concomitant diseases should be recognized and, if necessary, taken into account in a holistic treatment regimen. Based on the evaluated studies and the recently updated American/UK guidelines, the experts developed recommendations for the effective treatment of acne tarda (Table 4). Possible treatments for PIE/PIH and acne scars as well as dermocosmetics were also discussed (Table 5). These recommendations are intended to support dermatologists in offering their patients a holistic therapeutic approach.

## CONFLICT OF INTEREST STATEMENT

M.A. has been a consultant and/or paid speaker for companies and/or received institutional research funding and/or honoraria for consulting and/or scientific presentations and/or received travel reimbursements and/or participated in clinical trials sponsored by companies that manufacture compounds for the treatment of acne, including Almirall, Beiersdorf, Galderma, LEO, l'Oreal, Pierre Fabre, Roche Posay and Viatris. T.D. has received research funding from Almirall, Biofrontera, Galderma, Meda, Schulze & Böhm GmbH; consulting fees from Almirall, Biofrontera, GSK, Dr Pfleger, Galderma, Janssen‐Cilag, LEO, Meda, Neracare, Novartis, Scibase, smartinmedia AG, UCB and Vichy; and lecture fees from Almirall, Biofrontera, Galderma, GSK, Infectopharm, Janssen‐Cilag, LEO, Meda, Neracare, Novartis, Pfleger, Riemser, Scibase, smartinmedia AG, UCB and Vichy. P.A.G. is a consultant and investigator for Allergan, Galderma and Merz Aesthetics and has received honoraria as a speaker for Allergan, Croma, Galderma and Merz Aesthetics. M.K. declares no conflict of interest. N.K. has worked as a speaker and/or consultant for AbbVie, Eli Lilly, Janssen, Novartis, LEO Pharma, UCB and Uluru Inc. She has participated in clinical trials for AbbVie, Almirall, Boehringer Ingelheim, Eli Lilly, Janssen, Novartis, LEO Pharma, Pfizer, Sanofi and UCB. F.O. has worked as a consultant or received lecture fees from Galderma, GSK, Mylan and MSD.

## Supporting information



Supplementary information

Supplementary information
