# Supplementary material for: Acne tarda: Recommendations for classification, treatment and care as a result of an expert discussion
Source: J Dtsch Dermatol Ges. 2025 Dec 5;24(1):11–22. doi: 10.1111/ddg.15913 (PMC12800883; doi:10.1111/ddg.15913)
Supplement: Supplementary file 2 — Supplementary information [file DDG-24-11-s001.docx]

**Online supplementary table 2: Overview of clinical studies on treatment efficacy for acne tarda.** AFA = Adult female acne; AFAST-F = Adult Female Acne Scoring Tool - Face; EGSS = Evaluator's Global Severity Score; GAAS = Global Acne Assessment Score; IGA = Investigator's Global Assessment; ISGA = Investigator's Static Global Assessment; PGA = Physician's Global Assessment. Relevant studies, as of February 2025.

| **Authors** | **Study type, study group** | **Acne type** | **Intervention** |
| --- | --- | --- | --- |
| Gollnick H, et al. 1999^1^ | Open, multicentre study; n=890: women, 15-50 years of age | All types: open and closed comedones, papules, pustules, nodules, and cysts | 2 mg cyproterone acetate + 0.035 mg ethinyloestradiol |
| Dréno B, et al. 2009^2^ | Open, multicentre study; n=397: women aged 30-40 years | Acne, without further details | Retinaldehyde (0.1%) + glycolic acid (6%) |
| Rademaker M, et al. 2014^3^ | Randomised, placebo-controlled study; n=60: women and men, 22-55 years of age | Mild acne: ≥ 3 acne lesions/month on the face for ≥ 3 months; Grade 1-2 Modified Leeds Acne Assessment scale | Low-dose (5 mg) isotretinoin vs. placebo |
| Zeichner JA, et al. 2015^4^ | Post-hoc subgroup analysis; n=72: women, ≥25 years | Moderate to severe acne vulgaris: 20-40 papules/pustules/nodules, 20-100 open/closed comedones), ≤ 2 nodules | Clindamycin (1.2%) / benzoyl peroxide (3.75%) gel vs. vehicle |
| Thielitz A, et al. 2015^5^ | Randomised, monocentric, placebo-controlled study; n=55: women, 18-45 years of age | Mild to moderate acne: Grade 2-4 according to modified ISGA or 2-7 according to Leeds Revised Acne Grading Scale; ≤ 1 nodule | Azelaic acid (15%) gel vs. adapalene (0.1%) gel |
| Stein Gold L, et al. 2016^6^ | Meta-subgroup analysis of randomised, placebo-controlled studies; n=254: Women, ≥25 years | Mild, moderate, and severe acne after IGA | Adapalene (0.1%) / benzoyl peroxide (2.5%) gel vs. vehicle |
| Alexis AF, et al. 2016^7^ | Open, multicentre study; n=68: women, ≥18 years, coloured skin (Fitzpatrick types IV-VI) | Facial acne after GAAS | Dapsone (5%) gel |
| Kainz JT, et al. 2016^8^ | Prospective, non-interventional study; n=251: women aged ≥20 years | Mild to moderate acne (IGA grade 1-3) | Azelaic acid (20%) ointment |
| Harper JC, et al. 2019^9^ | Post-hoc subgroup analysis of 2 multicentre, randomised, placebo-controlled studies; n=606; women aged ≥18 years | Moderate to severe acne vulgaris according to EGSS | Tretinoin (0.05%) lotion vs. vehicle |
| Chottawornsak N, et al. 2019^10^ | Randomised, monocentric, placebo-controlled study; n=41: women, ≥25 years of age | Mild acne (AFA score 2: ≤50% of the face affected, few comedones, papules, pustules )^11^ | Ketoconazole (2%) ointment vs. vehicle |
| Patiyasikunt M, et al. 2020^12^ | Randomised, monocentric, placebo-controlled study; n=60: women, 25-45 years of age | Moderate acne grade 3 according to AFAST-F^13^ | Low-dose spironolactone (25/50 mg) + benzoyl peroxide 2.5% gel vs. vehicle |
| Chilicka K, et al. 2020^14^ | Randomised, monocentric study; n=120: women, 18-24 years of age | Mild to moderate papulopustular acne | Azelaic acid (16%) vs. pyruvic acid (50%) peeling |
| Cook-Bolden FE et al. 2020^15^ | Post-hoc subgroup analysis of 2 multicentre, randomised, placebo-controlled studies; n=268: men aged ≥18 years | Moderate to severe acne vulgaris: 20-50 papules/pustules/nodules, 25-100 open/closed comedones), ≤ 2 nodules | Tazarotene (0.045%) lotion vs. vehicle |
| Stein Gold L, et al. 2022^16^ | Post-hoc subgroup analysis of 2 multicentre, randomised, placebo-controlled studies; n=744: women ≥18 years of age | Moderate to severe acne vulgaris: 20-50 papules/pustules/nodules, 25-100 open/closed comedones), ≤2 nodules | Tazarotene (0.045%) lotion vs. vehicle |
| Gerber PA. 2023^17^ | Post-hoc subgroup analysis of a multicentre phase III long-term study; n = 46: women ≥ 25 years of age | Moderate acne: IGA/PGA = 3; ≥20 inflammatory lesions and ≥25 non-inflammatory lesions on the face; ≥20 inflammatory lesions and ≥20 non-inflammatory lesions on the trunk | Trifarotene (50 µg/g cream) |
| Santer M, et al. 2023^18^ | Multicentre, randomised controlled phase III study (UK, Wales), n=410; women aged ≥18 years | Facial acne for at least 6 months; IGA ≥2 | Spironlactone (50mg per day) or placebo, increased to 100 mg/day spironolactone or placebo after 6 weeks |
| Dréno B, et al. 2024^19^ | Multicentre, randomised, controlled, double-blind, prospective and parallel study (France), n=133; women aged ≥20 years | Moderate acne (at least 10 inflammatory lesions and no more than 3 nodules according to AFAST score on face and lower jaw) | Spironlactone (150 mg per day) vs. doxycycline (100 mg per day); both + 5% BPO |
| Baldwin H, et al. 2024^20^ | 2 multicentre, randomised, controlled, double-blind phase III studies with patients ≥9 years (n=363). Post hoc subgroup analysis for adult patients ≥ 18 years (n= 185) | Moderate to severe acne bach EGSS (grade 3 or 4); 30-100 facial inflammatory lesions; 35-150 noninflammatory lesions and ≤2 facial nodules. | Topical clindamycin phosphate 1.2%/ adapalene 0.15%/ benzoyl peroxide 3.1% gel (CAB; triple combination) vs. vehicle gel |

**References**

1. Gollnick H, Albring M, Brill K. [The effectiveness of oral cyproterone acetate in combination with ethinylestradiol in acne tarda of the facial type]. *Ann Endocrinol (Paris).* 1999;60:157-166.

2. Dréno B, Castell A, Tsankov N et al. Interest of the association retinaldehyde/glycolic acid in adult acne. *J Eur Acad Dermatol Venereol.* 2009;23:529-532.

3. Rademaker M, Wishart JM, Birchall NM. Isotretinoin 5 mg daily for low-grade adult acne vulgaris--a placebo-controlled, randomized double-blind study. *J Eur Acad Dermatol Venereol.* 2014;28:747-754.

4. Zeichner JA. The Efficacy and Tolerability of a Fixed Combination Clindamycin (1.2%) and Benzoyl Peroxide (3.75%) Aqueous Gel in Adult Female Patients with Facial Acne Vulgaris. *J Clin Aesthet Dermatol.* 2015;8:21-25.

5. Thielitz A, Lux A, Wiede A et al. A randomized investigator-blind parallel-group study to assess efficacy and safety of azelaic acid 15% gel vs. adapalene 0.1% gel in the treatment and maintenance treatment of female adult acne. *J Eur Acad Dermatol Venereol.* 2015;29:789-796.

6. Gold LS, Baldwin H, Rueda MJ et al. Adapalene-benzoyl Peroxide Gel is Efficacious and Safe in Adult Female Acne, with a Profile Comparable to that Seen in Teen-aged Females. *J Clin Aesthet Dermatol.* 2016;9:23-29.

7. Alexis AF, Burgess C, Callender VD et al. The Efficacy and Safety of Topical Dapsone Gel, 5% for the Treatment of Acne Vulgaris in Adult Females With Skin of Color. *J Drugs Dermatol.* 2016;15:197-204.

8. Kainz JT, Berghammer G, Auer-Grumbach P et al. Azelaic acid 20 % cream: effects on quality of life and disease severity in adult female acne patients. *J Dtsch Dermatol Ges.* 2016;14:1249-1259.

9. Harper JC, Baldwin H, Stein Gold L, Guenin E. Efficacy and Tolerability of a Novel Tretinoin 0.05% Lotion for the Once-Daily Treatment of Moderate or Severe Acne Vulgaris in Adult Females. *J Drugs Dermatol.* 2019;18:1147-1154.

10. Chottawornsak N, Chongpison Y, Asawanonda P, Kumtornrut C. Topical 2% ketoconazole cream monotherapy significantly improves adult female acne: A double-blind, randomized placebo-controlled trial. *J Dermatol.* 2019;46:1184-1189.

11. Dréno B, Poli F, Pawin H et al. Development and evaluation of a Global Acne Severity Scale (GEA Scale) suitable for France and Europe. *J Eur Acad Dermatol Venereol.* 2011;25:43-48.

12. Patiyasikunt M, Chancheewa B, Asawanonda P et al. Efficacy and tolerability of low-dose spironolactone and topical benzoyl peroxide in adult female acne: A randomized, double-blind, placebo-controlled trial. *J Dermatol.* 2020;47:1411-1416.

13. Poli F, Auffret N, Claudel JP et al. AFAST: an adult female acne treatment algorithm for daily clinical practice. *Eur J Dermatol.* 2018;28:101-103.

14. Chilicka K, Rogowska AM, Szygula R et al. A comparison of the effectiveness of azelaic and pyruvic acid peels in the treatment of female adult acne: a randomized controlled trial. *Sci Rep.* 2020;10:12612.

15. Cook-Bolden FE, Gold MH, Guenin E. Tazarotene 0.045% Lotion for the Once-Daily Treatment of Moderate-to-Severe Acne Vulgaris in Adult Males. *J Drugs Dermatol.* 2020;19:78-85.

16. Stein Gold L, Kircik L, Baldwin H et al. Tazarotene 0.045% Lotion for Females With Acne: Analysis of Two Adult Age Groups. *J Drugs Dermatol.* 2022;21:587-595.

17. Gerber PA. Akne – Bewährtes und Neues. *Thieme Kompendium Dermatologie.* 2023:28–34.

18. Santer M, Lawrence M, Renz S et al. Effectiveness of spironolactone for women with acne vulgaris (SAFA) in England and Wales: pragmatic, multicentre, phase 3, double blind, randomised controlled trial. *BMJ.* 2023;381:e074349.

19. Dréno B, Nguyen JM, Hainaut E et al. Efficacy of Spironolactone Compared with Doxycycline in Moderate Acne in Adult Females: Results of the Multicentre, Controlled, Randomized, Double-blind Prospective and Parallel Female Acne Spironolactone vs doxyCycline Efficacy (FASCE) Study. *Acta Derm Venereol.* 2024;104:adv26002.

20. Baldwin H, Gold LS, Harper JC et al. Triple-Combination Clindamycin Phosphate 1.2%/Adapalene 0.15%/Benzoyl Peroxide 3.1% Gel for Acne in Adult and Pediatric Participants. *J Drugs Dermatol.* 2024;23:394-402.
